# Worry is associated with inefficient functional activity and connectivity in prefrontal and cingulate cortices during emotional interference

**DOI:** 10.1002/brb3.1137

**Published:** 2018-10-30

**Authors:** Holly Barker, James Munro, Natasza Orlov, Elenor Morgenroth, Jason Moser, Michael W. Eysenck, Paul Allen

**Affiliations:** ^1^ Department of Psychology University of Roehampton London UK; ^2^ Department of Psychology Edinburgh Napier University Edinburgh UK; ^3^ Department of Psychosis Studies, Institute of Psychiatry, Psychology & Neuroscience King’s College London London UK; ^4^ Department of Psychology Michigan State University East Lansing Michigan; ^5^ Department of Psychology Royal Holloway University of London London UK; ^6^ Combined Universities Brain Imaging Centre London UK

**Keywords:** anterior cingulate, anxiety, dorsolateral prefrontal cortex, functional magnetic resonance imaging, worry

## Abstract

**Introduction:**

Anxiety is known to impair attentional control particularly when Task demands are high. Neuroimaging studies generally support these behavioral findings, reporting that anxiety is associated with *increased* (inefficient) activity in dorsolateral prefrontal cortex (DLPFC) and anterior cingulate cortex (ACC) during attentional control Tasks. However, less is known about the relationship between worry (part of the cognitive dimension of trait anxiety) and DLPFC/ACC function and connectivity during attentional control. In the present study, we sought to clarify this relationship.

**Methods:**

Forty‐one participants underwent functional magnetic resonance imaging (fMRI) during a composite Faces and Scenes Task with high and low emotional interference conditions. Individual worry levels were assessed using the Penn State Worry Questionnaire.

**Results:**

During high but not low emotional interference, worry was associated with increased activity in ACC, DLPFC, insula, and inferior parietal cortex. During high emotional interference, worry was also associated with reduced functional connectivity between ACC and DLPFC. Trait anxiety was not associated with changes in DLPFC/ACC activity or connectivity during either Task condition.

**Conclusions:**

The results are consistent with cognitive models that propose worry competes for limited processing resources resulting in inefficient DLPFC and ACC activity when Tasks demands are high. Limitations of the present study and directions for future work are discussed.

## 1 INTRODUCTION

Anxiety disorders are some of the most common psychiatric conditions in the western world (Remes, Brayne, Linde, & Lafortune, 2016). High levels of trait anxiety, a normally distributed personality dimension, can increase risk for anxiety disorders (Kessler, Chiu, Demler, Merikangas, & Walters, 2005; Taylor & Whalen, 2015). Moreover, it has been reported that trait anxiety can impair the ability to regulate attention (see Berggren & Derakshan, 2013 for review). Attentional Control Theory (ACT; Eysenck, Derakshan, Santos, & Calvo, 2007) proposes that high levels of trait anxiety compete for attentional resources and impair attentional control when executive processes are required, that is during cognitively demanding Tasks. A central prediction of ACT is that, when Tasks are demanding, anxiety can impair *processing efficiency* (the quality of performance relative to use of processing resources) to a greater extent than *performance effectiveness*. Performance effectiveness is largely maintained because anxious individuals can utilize compensatory processes to overcome conflict or interference, albeit inefficiently. Whilst ineffective and/or inefficient processing during behavioral Tasks (i.e., slower reaction times [RTs]) is not always observed in anxious individuals (Berggren & Derakshan, 2013), functional magnetic resonance imaging (fMRI) studies have reported *inefficient* neural processing*—*that is increased Task‐related activity without concomitant improved Task performance. Specifically, increased right (Basten, Stelzel, & Fiebach, 2011, 2012; Telzer et al., 2008) and bilateral (Fales et al., 2008; Karch et al., 2008; Telzer et al., 2008) dorsolateral prefrontal cortex (DLPFC) activity has been demonstrated in anxious individuals during Tasks requiring attentional control.

The fronto‐parietal network (FPN), particularly the DLPFC, is known to be important for attentional control (Braver, Paxton, Locke, & Barch, 2009; Sylvester et al., 2012) and supports “top‐down” attention by maintaining attentional sets (Braver et al., 2009; MacDonald, Cohen, Stenger, & Carter, 2000; Miller & Cohen, 2001). Increased anterior cingulate cortex (ACC) activity has also been reported in anxious individuals when Tasks require executive control (Comte et al., 2015; Etkin, Prater, Hoeft, Menon, & Schatzberg, 2010; Hajcak, McDonald, & Simons, 2003; Simmons et al., 2008). Increased ACC activity is thought to act as a compensatory mechanism in response to Task‐related conflict or interference (Sylvester et al., 2012). Furthermore, inefficient Task‐related DLPFC and ACC activation in high‐anxious individuals may be a consequence of suboptimal or reduced functional connectivity between these regions when increased attentional control is required (Basten et al., 2011; Comte et al., 2015). However, fMRI studies have also shown that trait anxiety is *not* associated with increased (inefficient) recruitment of frontal attentional control mechanisms (Bishop, 2009; Forster, Nunez Elizalde, Castle, & Bishop, 2015), a finding that appears inconsistent with the prediction made by ACT (Eysenck et al., 2007).

Conflicting fMRI findings could be due to the multidimensional nature of trait anxiety (Barlow, 1991). Specifically, it has been demonstrated that self‐reported anxiety can be decomposed into distinct physiological and cognitive dimensions that have different neuropsychological effects and correlates (Engels et al., 2007; Nitschke, Heller, Imig, McDonanld, & Miller, 2001). Whilst it has been demonstrated that worry is only one part of the cognitive dimension of trait anxiety (Grös, Antony, Simms, & McCabe, 2007), according to Processing Efficiency Theory (PET; Eysenck & Calvo, 1992), an earlier conceptualization of ACT, it is worry that competes for limited processing resources in anxious individuals occupying cognitive resources that would otherwise be allocated to attentional control (Eysenck & Calvo, 1992; Mathews, 1990; McNally, 1998).

Although fMRI has been widely used to investigate the effects of trait anxiety on attentional control, far fewer neuroimaging studies have directly examined the effects of worry on Task‐related brain activity. Engels et al. (2007) report that worry is associated with distinct patterns of brain activity in the frontal cortex during the presentation of threat stimuli. Worry also increases activity in the dorsal ACC to aid response selection (Silton et al., 2010) and is associated with delayed activation in attention‐related brain regions (Spielberg et al., 2013); findings seemingly consistent with the reduced processing efficiency prediction of PET. Electrophysiological studies also provide evidence that worry is associated with reduced neural efficiency during conflict monitoring Tasks (Moran, Bernat, Aviyente, Schroder, & Moser, 2015; Moser, Moran, Schroder, Donnellan, & Yeung, 2013).

In the present study, we aimed to clarify the effects of worry on Task‐related activity in the DLPFC and ACC; brain regions involved in attentional control. We predicted that the cognitive process of worry would compete for neural resources to a greater extent than the more general construct of trait anxiety. We used an fMRI Task with conditions of high and low emotional interference, as the presence of emotionally salient distractors leads to competition for attentional resources (Klumpp et al., 2011). In accordance with the predictions of PET (Eysenck & Calvo, 1992), we hypothesized that worry, rather than trait anxiety, would be associated with increased (inefficient) DLPFC/ACC activity and reduced functional connectivity between these regions. In accordance with ACT, we predicted that the association between worry and increased DLPFC and ACC activity/connectivity would be seen during high but not low emotional interference.

## 2 METHODS

### 2.1 Participants and assessments

Forty‐nine participants were recruited to the fMRI study; however, eight participants had incomplete data sets (four due to incomplete fMRI data and four due to missing questionnaire data); thus, all analyses are based on 41 participants. Participants (27 female) ranged from 18 to 37 years of age (*M* = 22.53 years, *SD* = 4.63). There were 35 right‐handed and six left‐handed participants, as measured by the Annett Hand Preference Questionnaire (Annett, 1970). Ethical approval was granted by the University of Roehampton, London, UK, and all participants gave informed written consent before taking part in the study. Participants self‐reported no present or prior history of psychiatric or neurological illness and no contraindication for MRI. The Wide Range Achievement Test (WRAT‐R) Reading Level 2 (Jastak & Wilkinson, 1984) was used to estimate IQ. Estimated IQ scores ranged from 91 to 131 (*M* = 111.70, *SD* = 10.02). The Penn State Worry Questionnaire (PSWQ; Meyer, Miller, Metzger, & Borkovec, 1990) was used to measure anxious apprehension (i.e., worry), and the State‐Trait Anxiety Inventory (STAI; Spielberger, Gorsuch, Lushene, Vagg, & Jacobs, 1983) was used to measure state and trait anxiety. Participants’ worry levels (PSWQ scores) ranged from 23 to 79 (*M* = 50.21, *SD* = 14.57, Cronbach's alpha = 0.95). STAI trait anxiety levels ranged from 24 to 59 (*M* = 40.76 *SD* = 9.60, Cronbach's alpha = 0.93), and state anxiety levels ranged from 20 to 54 (*M* = 33.09 *SD* = 8.50; Cronbach's alpha = 0.95).

### 2.2 Experimental Task

An adapted version of the Composite Faces/Scenes Task (CFST; Anderson, Christoff, Panitz, Rosa, & Gabrieli, 2003; Klumpp et al, 2011) was used in the fMRI paradigm. The CFST requires sustained attention and comprises gray‐scale images of Faces (Fearful/Neutral emotional expression) superimposed onto images of Neutral Scenes (indoor/outdoor normalized to a mean gray value of 127), so that they appeared within the same visual field with 50% transparency of each image During the CFST, participants were instructed to Attend to one element of the composite image, either Faces or Scenes. During the Attend Face condition, participants were instructed to make judgments about the gender of the Face. This constituted a high emotional interference condition as we expected participants to be distracted by the emotional expression of Fearful Faces. During the Attend Scene condition, participants were instructed to ignore Faces and make judgments about the Scene location (indoor/outdoor); thus, interference from emotional Faces (the to‐be‐ignored stimuli) was expected to be low. Composite stimuli comprised 32 images of Faces (16 Fearful and 16 Neutral, each with eight male and eight female Faces) sourced from the Nimstim Face database (https://www.macbrain.org/resources.htm; Tottenham et al., 2009), superimposed over 64 images of Scenes (32 indoor Scenes and 32 outdoor Scenes), using Photoshop CS6 software. There were 64 original gray‐scale images in total. Each trial contains a composite Face/Scene image presented for 2 s. The trial structure follows a response inhibition Task design: 28/32 of trials contained the target subcategory (e.g., an indoor Scene after an indoor cue) and requires a response (button press); the other 4/32 contains the non‐target subcategory, that is (an outdoor Scene after an indoor cue) to which responses must be withheld.

During fMRI, participants were presented with four CFST conditions: two conditions for “Attend Face” (one requiring a response to male Faces/one requiring a response to female Faces) and two conditions for “Attend Scene” (one requiring a response to indoor Scenes/one requiring a response to outdoor Scenes). Each condition consisted of 32 trials, with 16 Neutral and 16 Fearful Faces present. Attend Face/Scene conditions were counterbalanced across subjects. Participants were instructed to respond via a button press if these features (male/female or indoor/outdoor) were present but to withhold a response if the feature was not present. During each condition, four non‐target trials were presented in which the non‐target feature was present (e.g., an outdoor Scene after an instruction to Attend to indoor Scenes); participants were required to withhold their response. Each trial was presented for 2 s with a randomized inter‐stimulus interval of either 2, 4, 6, or 8 s and a fixation cross presented between trials. E‐prime (Psychology Software Tools, Pittsburgh, PA) software was used to present the Task stimuli and collect RTs and accuracy data. The Task took approximately 18 min to complete.

### 2.3 MRI acquisition

Scanning was performed using a 3T Siemens Magnetom TIM Trio scanner with a Siemens 32‐channel head array coil. Structural images were obtained using a T1‐weighted Magnetization Prepared Rapid Acquisition Gradient Echo sequence (1 mm × 1 mm × 1 mm). Functional images were acquired using a full‐brain, anterior‐to‐posterior, T2*‐weighted, BOLD‐sensitive gradient echo‐planar sequence (Repetition time = 2,000 ms, Echo time = 40 ms, Voxel Size = 3 mm × 3 mm × 5 mm, Field of View = 192 mm^2^, Flip Angle = 70°, Slice thickness = 5 mm [with no inter‐slice gap]).

### 2.4 Behavioral analysis

IBM® SPSS Statistics Version 22 was used for the analysis of behavioral data. Data were subjected to normality tests and all accuracy variables were transformed using a Reverse Log10 transformation and back‐transformed to report results. RTs and accuracy data were analyzed using a 2 (Task: Attend Face/Attend Scene) × 2 (Face: Neutral/Fearful) two‐way repeated measures analysis of covariance, with PSWQ and STAI trait anxiety as covariates of interest. A statistical significance threshold of *p *< 0.05 was applied throughout. Post hoc Pearson product‐moment correlations were performed on data to test the association between PSWQ scores and RT/accuracy data.

### 2.5 fMRI data analysis

Statistical Parametric Mapping (version 12; https://www.fil.ion.ucl.ac.uk/spm/) software running on Matlab R2016a was used to analyze fMRI data. Structural and functional images were manually reoriented to the anterior commissure–posterior commissure line so that they matched the normalized space that the images would later be normalized to. Images were realigned using rigid body transformations, using the six movement parameters in order to reduce movement artefacts. Functional images were then co‐registered to anatomical images for each participant to facilitate clear visualization of results. Segmentation was carried out and images were normalized to the Montreal Neurological Institute template to allow for group comparisons. Functional images were spatially smoothed using an 8 mm Gaussian kernel. A general linear model (GLM) was used to model data from the CFST using the following regressors: Attend Face (Fearful Face trials), Attend Face (Neutral Face trials), Attend Scene (Fearful Face trials), and Attend Scene (Neutral Face trials). Fixation cross trials served as an implicit baseline. The six movement parameters were modeled as regressors of no interest, in order to further reduce movement artefacts. To reduce low‐frequency noise, a high‐pass filter was applied with a cutoff of 128 s.

To examine the effect of high versus low emotional interference on neural activity, the first level contrasts (Attend Face Fearful > Attend Face Neutral) and (Attend Scene Fearful > Attend Scene Neutral) were specified and entered into second‐level paired *t* tests. To examine the effects of worry (PSWQ scores) on brain activation during high and low emotional interference conditions, first level contrast images (Attend Face Fearful > Attend Face Neutral) and (Attend Scene Fearful > Attend Scene Neutral) were entered into separate second‐level multiple regression models with PSWQ scores as a covariate of interest and behavioral performance (accuracy), as a covariate of no interest to account for the effects of performance on variance within the model. Gender and estimated IQ scores were also entered as covariates of no interest. These second‐level multiple regression models were repeated with STAI trait anxiety scores, to test if effects were specifically related to worry, or the more general construct of trait anxiety. A significance threshold of *p *< 0.05 (Family Wise Error‐cluster level) set at a peak‐level cluster detection threshold of *p *< 0.001 was used throughout the study and only brain activation meeting this significance threshold was reported (Eklund, Nichols, & Knutsson, 2016; Woo, Krishnan, & Wager, 2014).

### 2.6 Psychophysiological interaction analysis

Psychophysiological interaction (PPI) analyses (Friston et al., 1997) were conducted to examine if worry (PSWQ scores) modulated Task‐specific functional coupling of ACC with the DLPFC. Based on greater ACC activation during the emotional interference Task, the ACC was chosen as a seed region for PPI analysis. Seed coordinates were determined from the coordinates of peak activation in the contrast (Attend Face Fearful > Attend Face Neutral) > (Attend Scene Fearful > Attend Scene Neutral) which elicited functional activity in right cingulate gyrus/medial frontal gyrus (8, 30, 30) at the group level. Subject‐specific eigenvariate time series of the BOLD signal were extracted from a 6 mm sphere around the seed coordinates using the effects of interest. The GLMs for each participant consisted of this physiological regressor (the extracted time series), the psychological regressor (Attend Face vs. Attend Scene), and the PPI regressor (the interaction of the psychological and physiological regressors), and the six movement parameters were modeled as regressors of no interest. At the group level, the PPI contrast images were entered into a second‐level random effects regression model with worry (PSWQ scores) as a covariate of interest and behavioral performance (accuracy), gender and IQ scores as covariates of no interest. For the analysis based on the ACC seed region, 40 participants were entered into the regression model (nine participants were excluded due to missing data/no suprathreshold activation in the seed region).

Based on our a priori hypothesis, we chose to examine Task‐specific functional coupling between the ACC seed region and the bilateral DLPFC. We defined the central points and spatial extent of DLPFC ROIs using an 8‐mm radius sphere. The central coordinates for the DLPFC ROIs were derived from reviews of Tasks manipulating attentional control (Duncan & Owen, 2000) and those used in a previous fMRI study of attentional control and trait anxiety (Bishop, 2009; central coordinates: ±34, 36, 24; included parts of the bilateral middle frontal gyrus and inferior frontal sulcus). Results are reported at a significance level of *p *< 0.025 FWE peak level to account for two tests (i.e., left/right DLPFC ROIs).

## 3 RESULTS

### 3.1 Behavioral results

**Figure 1:**
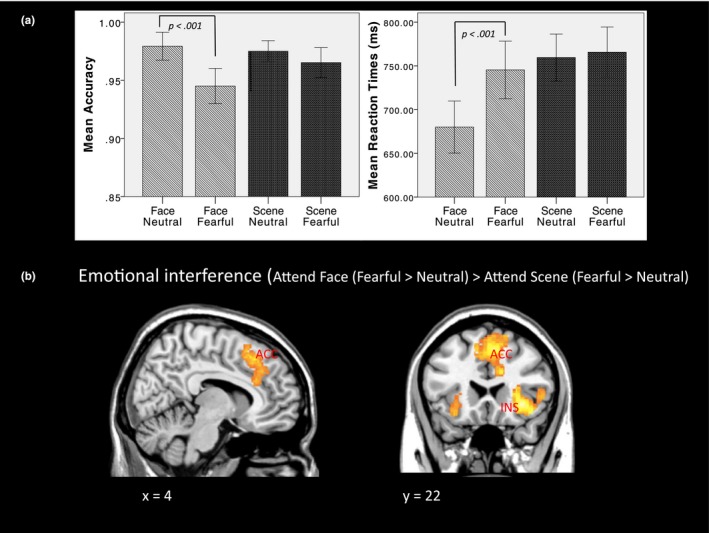
(a) Bar charts showing behavioral data during the Attend Face and Attend Scene conditions (accuracy and reaction times), (b) Statistical Parametric Mapping (SPM) showing main effect of emotional interference (Attend Face [Fearful > Neutral Face trials) > Attend Scene [Fearful > Neutral trials); INS = Insula, ACC = anterior cingulate cortex). The left side of the brain is shown on the left hand side of the image

Means and standard deviations for accuracy and RT data are shown in Figure 1a. All participants performed the Task with a high degree of accuracy (i.e., proportion of correct responses). For accuracy data, the main effect of Tasks (i.e., Attend Faces vs. Attend Scenes is reported in Supporting Information) was non‐significant. However, the Task × Face interaction effect was significant, *F*(1,40) = 17.81, *p *< 0.001. During the Attend Face condition, gender judgments were less accurate when Fearful Faces were present compared to Neutral Faces, *t*(40) = −5.02 *p *< 0.001. In the Attend Scene condition, accuracy for making place judgments was not affected by the emotion type of the to‐be‐ignored Face stimuli. For RT data, a significant Task × Face interaction term, *F*(1,40) = 59.93, *p *< 0.001, revealed that in the Attend Face condition, participants were slower to respond when Fearful Faces were presented compared to Neutral Faces, *t*(40) = −9.90, *p *< 0.001. When making place judgments in the Attend Scene Task, RTs were not effected by the emotional content of the to‐be‐ignored Face stimuli. When PSWQ scores were entered into the model as a covariate, there was a trend level effect on the Task × Face × PSWQ interaction term for RT, *F*(1,38) = 3.66 *p* = 0.063. There was a trend correlation between PSWQ scores and the increase in RT for Fearful > Neutral Faces in the Attend Face condition (*r* = 0.29, *p* = 0.062). The effect of PSWQ scores on accuracy was non‐significant. The effect of STAI trait anxiety scores on accuracy and RT was also non‐significant.

### 3.2 fMRI results

#### 3.2.1 High versus low emotional interference

Relative to the Attend Scene condition (Fearful > Neutral Faces), the Attend Face condition (Fearful > Neutral Faces) activated the right insula/claustrum extending to the frontal operculum (*x*,* y*,* z* = 32, 28, −16, *Z* = 4.44, *K*_E_ = 801, *P*_FWE_ **< 0.001), the right anterior cingulate gyrus/medial frontal gyrus (x, y, z = 8, 30, 30, Z = 4.18, *K*_E_ = 1,002, *P*_FWE_ **< 0.001), and the right angular gyrus/intraparietal sulcus (*x*,* y*,* z* = 52, −48, 42, *Z* = 4.13, *K*_E_ = 364, *P*_FWE_ = 0.002; Figure 1b). There was no suprathreshold effect for the contrast Attend Scene (Fearful > Neutral Faces) > Attend Face (Fearful > Neutral Faces).

#### 3.2.2 Effects of worry

**Figure 2:**
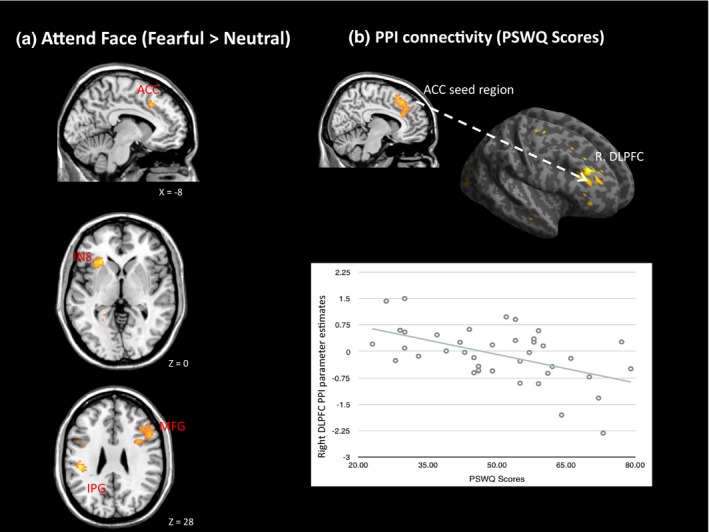
(a) Statistical Parametric Mapping (SPM) showing effects of worry (Penn State Worry Questionnaire [PSWQ] scores positive association) on regional activation during the Attend Face condition, (b) PSWQ regression on functional connectivity during Attend Face Condition, with scatter plot showing PPI parameter estimates (functional connectivity) against PSWQ scores. (ACC = anterior cingulate cortex; INS = insula; IPG = inferior parietal gyrus; MFG = middle frontal gyrus). Left side of the brain is shown on the left side of the image

During the Attend Face condition (Fearful > Neutral Faces), PSWQ scores were positively associated with activity in the left inferior parietal lobe (*x*,* y*,* z* = −44, −28, 28, *Z* = 4.21, *K*_E_ = 1934, *P*_FWE_ **< 0.001), the left cingulate gyrus/superior frontal gyrus (*x*,* y*,* z* = −10, 0, 30, Z = 3.51, *K*_E_ = 388, *P*_FWE_ **< 0.001), the right middle frontal gyrus (*x*,* y*,* z* = 38, 28, 22, Z = 3.25, *K*_E_ = 547, *P*_FWE_ = 0.002), and the left insula/claustrum (*x*,* y*,* z* = −28, 24, 4, *Z* = 4.14, *K_E_* = 281, *P*_FWE_ = 0.001; Figure 2a). During the Attend Scene condition (Fearful > Neutral Faces), there was no suprathreshold activation associated with PSWQ scores. When STAI trait anxiety scores were entered into multiple regression models, there were no suprathreshold effects during the Attend Face nor Attend Scene conditions.

#### 3.2.3 Functional connectivity

Penn State Worry Questionnaire scores predicted Task‐specific functional connectivity between the ACC seed region and the right middle frontal gyrus within the DLPFC ROI (*x*,* y*,* z* = 30, 34 20, *Z* = 3.6, *K*_E_ = 14, *P*_FWE (SVC)_ = 0.012). The right middle frontal gyrus showed significantly weaker Task‐specific functional coupling with the ACC during the Attend Face condition (Figure 2b). There were no effects of PSWQ scores on functional connectivity in the Attend Scene condition. STAI trait anxiety scores did not predict functional connectivity between the ACC seed region and DLPFC during either Task condition.

## 4 DISCUSSION

The aim of the present study was to examine the relationship between worry and DLPFC/ACC activity and functional connectivity during a Task in which levels of emotional interference could be altered whilst holding Task stimuli constant. Consistent with previous findings (Anderson et al., 2003; Klumpp et al., 2011), behavioral data showed that during the Attend Face condition (gender judgments), the emotion of the Face type affected both accuracy and RTs, with significantly reduced accuracy and slower RTs for Fearful relative to Neutral Faces. As expected, in the Attend Scene condition (location judgments), accuracy and RTs were not affected by the emotional content of the Task irrelevant (to‐be‐ignored) Face stimuli. These behavioral results suggest that Fearful facial stimuli increased competition for attentional resources and interfered with the Task of identifying Face gender. Conversely, when attention was directed away from Faces toward Scenes there was less interference from Fearful relative to Neutral Face stimuli. However, neither performance accuracy nor RTs were significantly affected by worry or trait anxiety, a finding consistent with some previous behavioral studies (Berggren & Derakshan, 2013).

Functional magnetic resonance imaging results showed that participants activated the right anterior cingulate, insula/operculum, and right angular gyrus during the Attend Face condition when Fearful Faces were presented (high emotional interference). The cingulate and insular/opercular network, sometimes referred to as the salience network (Seeley et al., 2007), is important for detecting conflict or interference and signaling the need for increased attentional control (Botvinick, Braver, Barch, Carter, & Cohen, 2001; Carter, Botvinick, & Cohen, 1999), possibly by relaying information to regions in the FPN (Moran et al., 2015; Sylvester et al., 2012). Whilst there was a significant reduction in Task performance in the Attend Face condition (i.e., reduced accuracy and slower RT during Fearful Face trials), overall performance effectiveness in this condition was still high (i.e., mean accuracy of around 95%), suggesting that increased right ACC/insula activation may have maintained effective Task performance under conditions of emotional interference. During the Attend Scene condition, the presence of Fearful Faces (relative to Neutral Faces) was not associated with activation in DLPFC or ACC (no suprathreshold activation in any region was observed), suggesting that during the low emotional interference condition, there was little or no interference to detect and relay.

It has been proposed that worry, a cognitive dimension of trait anxiety, co‐opts available cognitive resources that would otherwise be allocated to the Task at hand leading to inefficient Task processing (Eysenck & Calvo, 1992) particularly when Task demands are high (Eysenck et al., 2007). Although no affect on Task performance was observed, during the Attend Face condition (Fearful > Neutral Faces), worry, but not trait anxiety, was associated with increased activity in the left ACC, insula, inferior parietal gyrus, and the right middle fontal gyrus. Given that individual difference in worry did not significantly affect measures of Task accuracy or RT, these results suggest that increased neural activity in these regions was required to maintain adequate Task performance. This finding supports the prediction that under conditions of high emotional interference, whilst not effecting Task performance measures, worry is associated with inefficient neural activation in DLPFC and ACC regions. Conversely, during the Attend Scene condition there were no regions where functional activation was associated with worry or trait anxiety, suggesting that under conditions of low emotional interference, no additional neural resources were required.

Our functional findings appear consistent with the predictions of Eysenck and Calvo (1992) and replicate previous fMRI studies reporting that worry is associated with increased neural activity in regions important for attentional control (Silton et al., 2010; Spielberg et al., 2013). Here, we extend these previous findings by demonstrating that worry (a cognitive dimension of trait anxiety) impairs neural processing efficiency in attentional networks (Eysenck et al., 2007), specifically when Tasks are cognitively demanding. Thus, it is possible that worry can be maladaptive in terms of neural processing efficiency in DLPFC and ACC although such maladaptive effects did not extend to behavioral efficiency or effectiveness.

Functional connectivity analysis showed that, in the Attend Face condition, worry was associated with reduced coupling between the right ACC and middle frontal gyrus (DLPFC). Although reduced ACC‐DLPFC functional connectivity in high trait anxiety has been reported previously (Basten et al., 2011), no fMRI studies have investigated the relationship between worry and ACC‐DLPFC connectivity; although an association between worry and reduced ACC‐DLPFC connectivity has been reported using an EEG‐based phase synchrony metric (Moran et al., 2015). ACC‐DLPFC coupling, and the transmission of information between these regions, is thought to be important for executive control processes (Zanto & Gazzaley, 2013), particularly for detecting Task conflict/interference and then updating the DLPFC so attentional control can be maintained (Basten et al., 2011; Moran et al., 2015). Thus, worry may impair the ability of the DLPFC to update attentional set. Consequently, the DLPFC may have to rely on “reactive” (Braver, 2012) control strategies such as inhibition to maintain attentional control. Increased reliance on reactive control mechanisms to maintain Task performance could constitute a form of neural inefficiency.

However, the results of the present study appear inconsistent with a previous findings reporting that, during an inhibition Task, altered recruitment of frontal attentional control mechanisms was unrelated to worry (Forster et al., 2015). It is unclear why the results of this study differ from the present findings but it is possible that the different fMRI Task paradigms used in the Forster et al. and present study contributed to these discrepant findings. This study also had a considerably smaller sample size (*N* = 18) than the present study. However, Forster et al. (2015) did report a relationship between worry and greater DLPFC‐precuneus and DLPFC‐posterior cingulate connectivity (a posterior regions in the Default Mode Network; DMN), indicative of increased off‐task thought. There is increasing evidence that worry and mind wandering both involve the DMN, and that worry is associated with high DMN activation at rest (Fox, Spreng, Ellamil, Andrews‐Hanna, & Christoff, 2015; Servaas, Riese, Ormel, & Aleman, 2014) and during Tasks (Maresh, Allen, & Coan, 2014). Moreover, Task‐related deactivation of DMN regions is important for effective Task performance (Weissman, Roberts, Visscher, & Woldorff, 2006). Interestingly, in addition to increased DLPFC and ACC activity, we also observed increased left inferior parietal lobe activation associated with worry in the high emotional interference condition. The left inferior parietal lobe has been shown to be activated by language processes (Price, 2010) and may reflect activity related to the covert verbal nature of worry, a process that may compete for limited cognitive resources.

### 4.1 Limitations

It is possible that fMRI's poor temporal resolution conflates the strength of goal set prior to Task presentation with processes during Task performance itself. There is evidence that individuals high in anxiety or worry have a reduced ability to maintain Task goals prior to Task presentation (Braver, 2012). This would likely be the case when several inter‐trial intervals are used in a random fashion. An implication is that there needs to be more focus on FPN activation immediately prior to Task presentation on each trial. Also, whilst the vast majority of trials did not require motor inhibition, it is possible that neural effects seen during the high emotional interference condition (i.e., increased ACC and bilateral insula activity) were partly driven by trials requiring a withheld motor response (i.e., non‐target trials) as motor inhibition under conditions of high emotional interference may require greater neural resources. Furthermore, in our sample we were unable to control for visceral state effects that may also compromise cognition and/or attentional control (e.g., Lewis et al., 2011). Second, because the sample was non‐clinical, it is difficult to extend our findings to clinical populations such as those with generalized anxiety disorder or post‐traumatic stress disorder. Future work could extend the fMRI paradigm to psychiatric patients. Next, the concept of neural efficiency/inefficiency that is central to PET and ACT does not tell us about the precise neural mechanisms that underlie the different patterns of brain activation in people with high levels of worry. For example, differences in intensity and timing of neural signaling (i.e., temporal dynamics) as well as resting cerebral blood flow and metabolism would be likely to affect activation in fMRI experiments (Poldrack, 2015). How these factors affect the BOLD signal in people with high levels of worry needs to be the focus of future imaging studies. Finally, a further limitation of the current study is the failure to properly dissociate cognitive and physiological dimensions of trait anxiety. Future work could use an instrument such as the State‐Trait Inventory for Cognitive and Somatic Anxiety (Grös et al., 2007) which would have allowed us to tease apart trait anxiety's components, and how these differentially affect neural activity in the DLPFC and ACC.

## 5 CONCLUSIONS

Our fMRI results support the predictions of PET, that is that worry co‐opts processing resources. Although worry did not affect Task performance (accuracy and RTs), worry, rather than trait anxiety, was associated with increased (inefficient) Task‐related activity in the DLPFC and ACC. This finding may provide nuance to ACT by demonstrating that a cognitive dimension accounts for neural processing inefficiency rather than the more nebulous construct of trait anxiety. Moreover, and consistent with the predictions of ACT, worry was associated with inefficient neural activation in the DLPFC and ACC during high, but not low emotional interference. Worry also reduced functional connectivity between the ACC and DLPFC under conditions of high emotional interference. Future work is needed to further investigate the neural mechanisms that underlie neural processing inefficiency in people with high levels of worry, particularly to track the time course of DLPFC/ACC activation before and during Task performance.
