# Supplementary material for: Worry is associated with inefficient functional activity and connectivity in prefrontal and cingulate cortices during emotional interference
Source: Brain Behav. 2018 Oct 30;8(12):e01137. doi: 10.1002/brb3.1137 (PMC6305912; doi:10.1002/brb3.1137)
Supplement: Supplementary file 1 [file BRB3-8-e01137-s001.docx]

**TITLE**: Worry is associated with inefficient functional activity and connectivity in prefrontal and cingulate cortices during emotional interference

**AUTHORS:** Holly Barker MSc ^1^, James Munro PhD ^1, 2^ Natasza Orlov PhD ^1, 3^ Elenor Morgenroth MSc ^1^, Michael W. Eysenck PhD ^1,4^, Paul Allen PhD ^1,3, 5^

**Supplementary Material**

Affiliations

1. Department of Psychology, University of Roehampton, London, United Kingdom (UK)
2. Department of Psychology, Edinburgh Napier University, Edinburgh, UK
3. Department of Psychosis Studies, Institute of Psychiatry, Psychology & Neuroscience, King’s College London, London, UK
4. Department of Psychology, Royal Holloway University of London, London UK
5. Combined Universities Brain Imaging Centre, London, UK

Corresponding author:

Paul Allen

Department of Psychology, University of Roehampton, Whitelands College, Hollybourne Avenue, London SW15 4JD

Tel: 0044 (0)2083925147

Email: [paul.allen@roehampton.ac.uk](mailto:paul.allen@roehampton.ac.uk)

**RESULTS**

**STAI and PSWQ distributions**

For both PSWQ and STAI-trait anxiety scores skewness < 1 and Kurtosis was < 3 (figure s1).


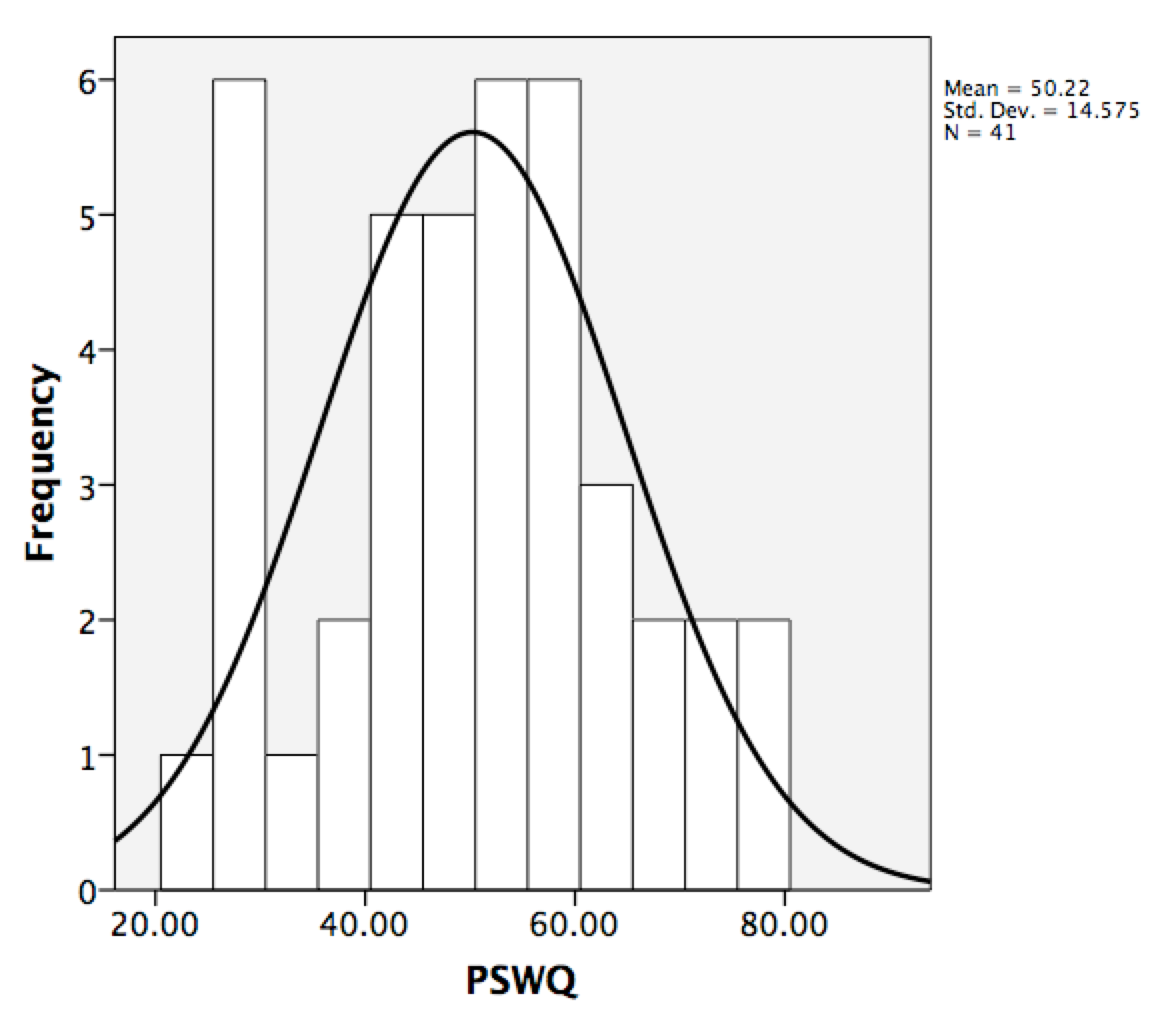

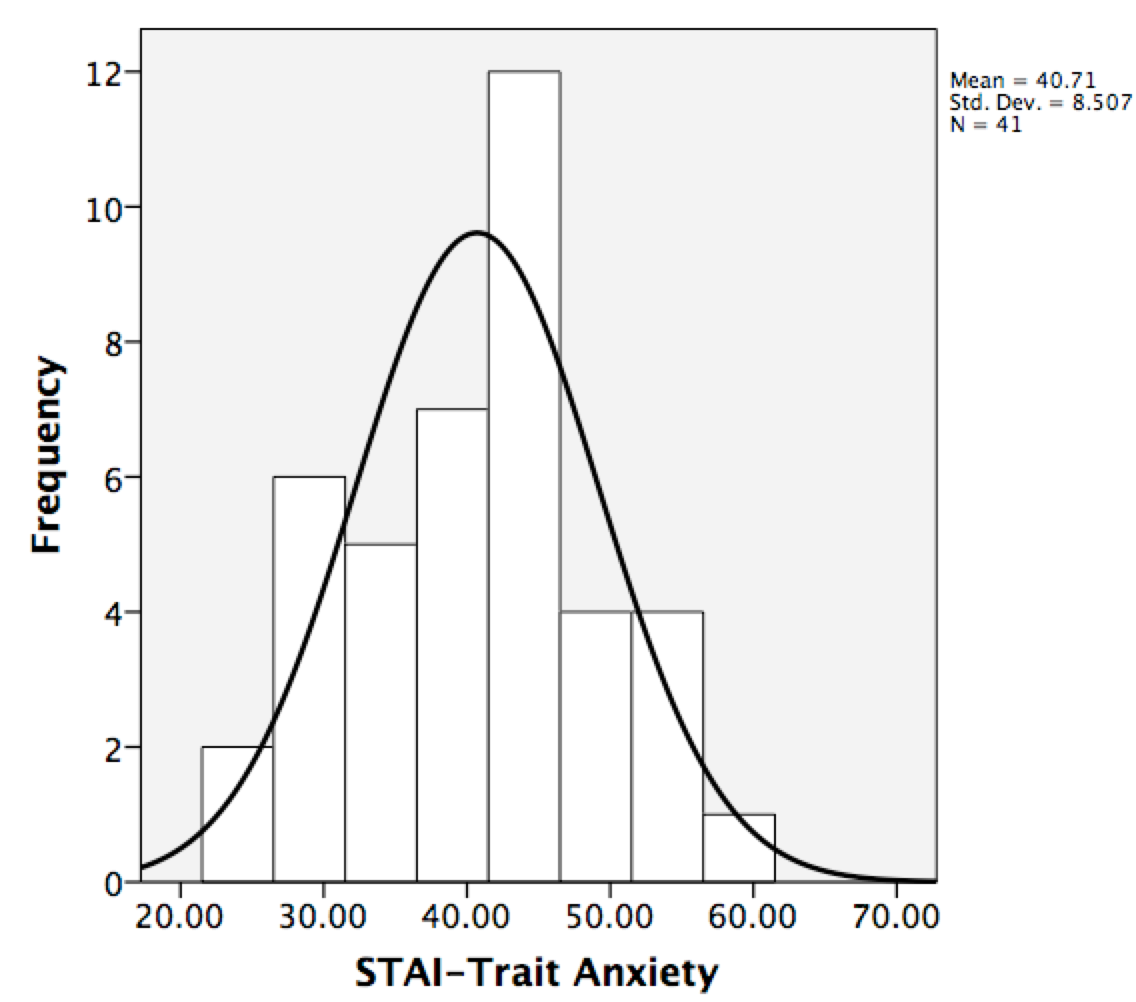


**Figure s1**. Histograms showing case frequency distribution for STAI trait anxiety and PSWQ scores


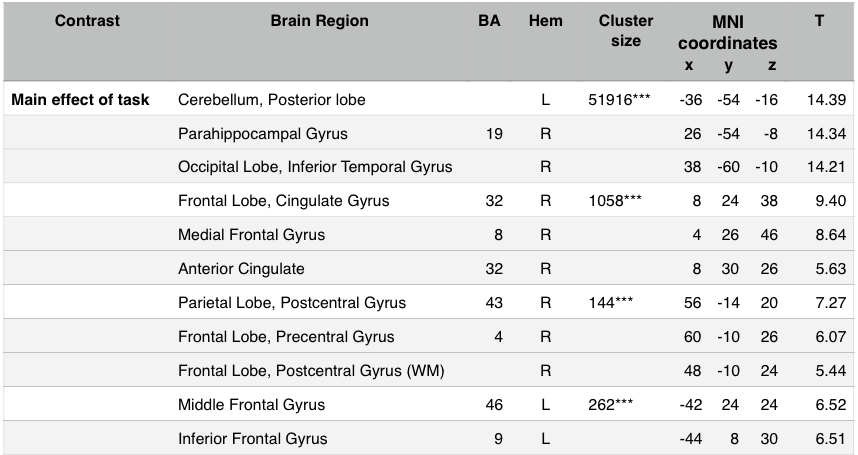


**Table s1:** Brain activation for main effect of task vs. baseline. BA = Brodmann area, Hem = hemisphere (Left/Right).  *** Significant at peak-level *p*FWE-corrected <0.05


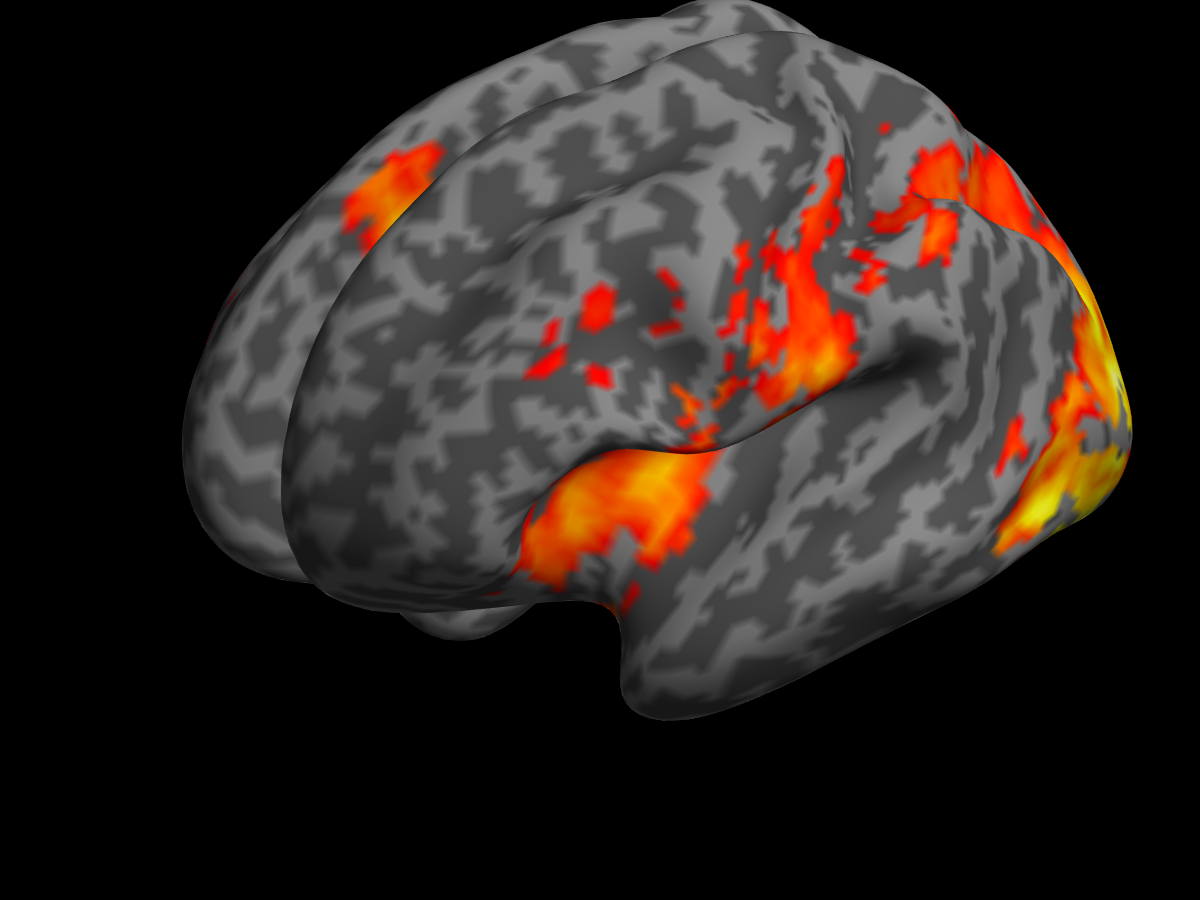

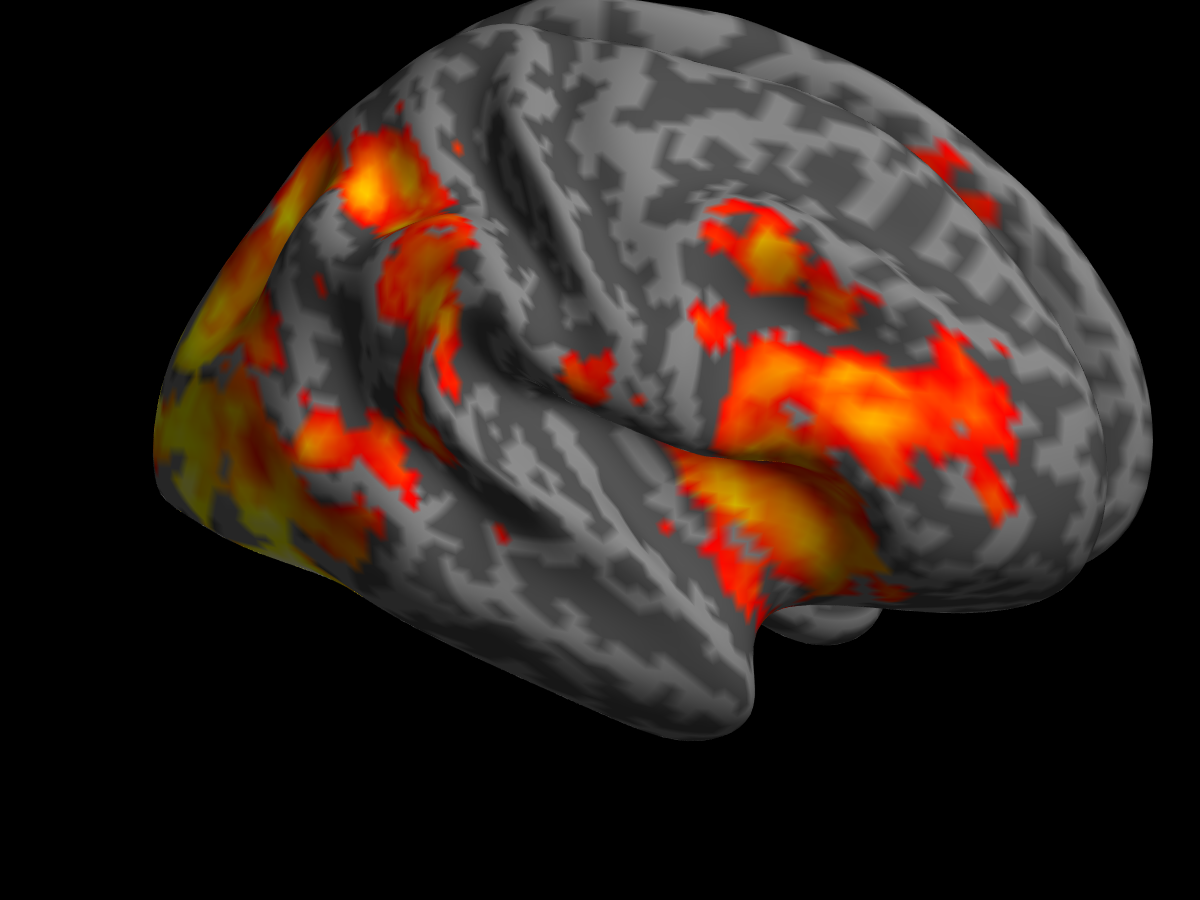


**Figure s2:** Statistical Parametric Maps (renders) of main effect of task in left and right hemispheres (peak-level *p*FWE-corrected <0.05)

Main Effects of Task (attend Faces vs. attend Scenes Task): When participants attended to Faces (Faces > Scenes), a cluster of activity was seen in the bilateral medial prefrontal cortex in the superior frontal gyri extending to the middle frontal gyrus and in the left thalamus (right x, y, z=2, 54, 32, Z=4.20; left x, y, z=-6, 54, 24, Z=4.07; *K_E=_*599, *P*_FWE_<.001). When participants attended to Scenes (Scenes > Faces) increased activation was seen in the bilateral parahippocampal gyrus extending to the lingual gyrus (right x, y, z=32, -46, 6, Z=6.24, *K_E_*=883, *P*_FWE_<.001; left x, y, z=-30, -50, -6, Z=6.46, *K_E_*=894, *P*_FWE_<.001), the right middle occipital gyrus extending to the angular gyrus (x, y, z=26, -80, 14: Z=4.90, *K_E_*=2852, *P*_FWE_<.001) and left precuneus/superior parietal lobule extending to the left occipital lobe (x, y, z=-14, -62, 54, Z=4.41, *K_E_*=2205, *P*_FWE_<.001).
